# Mitochondrial RNA in Alzheimer’s Disease Circulating Extracellular Vesicles

**DOI:** 10.3389/fcell.2020.581882

**Published:** 2020-11-16

**Authors:** Kyoung Mi Kim, Qiong Meng, Olivia Perez de Acha, Maja Mustapic, Aiwu Cheng, Erden Eren, Gautam Kundu, Yulan Piao, Rachel Munk, William H. Wood, Supriyo De, Ji Heon Noh, Michael Delannoy, Lesley Cheng, Kotb Abdelmohsen, Dimitrios Kapogiannis, Myriam Gorospe

**Affiliations:** ^1^Laboratory of Genetics and Genomics, National Institute on Aging Intramural Research Program, National Institutes of Health, Baltimore, MD, United States; ^2^Department of Biological Sciences, Chungnam National University, Daejeon, South Korea; ^3^Laboratory of Clinical Investigation, National Institute on Aging Intramural Research Program, National Institutes of Health, Baltimore, MD, United States; ^4^Department of Biochemistry, Chungnam National University, Daejeon, South Korea; ^5^Department of Cell Biology and Imaging Facility, Johns Hopkins University School of Medicine, Baltimore, MD, United States; ^6^Department of Biochemistry and Genetics, School of Molecular Science, La Trobe University, Melbourne, VI, Australia

**Keywords:** extracellular vesicles, Alzheimer’s disease, mitochondrial protein, mitochondrial RNA, biomarker

## Abstract

Alzheimer’s disease (AD) is the most common type of dementia. Amyloid β (Aβ) plaques, tau-containing neurofibrillary tangles, and neuronal loss leading to brain atrophy are pathologic hallmarks of AD. Given the importance of early diagnosis, extensive efforts have been undertaken to identify diagnostic and prognostic biomarkers for AD. Circulating extracellular vesicles (EVs) provide a platform for “liquid biopsy” biomarkers for AD. Here, we characterized the RNA contents of plasma EVs of age-matched individuals who were cognitively normal (healthy controls (HC)) or had mild cognitive impairment (MCI) due to AD or had mild AD dementia (AD). Using RNA sequencing analysis, we found that mitochondrial (mt)-RNAs, including *MT-ND1-6* mRNAs and other protein-coding and non-coding mt-RNAs, were strikingly elevated in plasma EVs of MCI and AD individuals compared with HC. EVs secreted from cultured astrocytes, microglia, and neurons after exposure to toxic conditions relevant to AD pathogenesis (Aβ aggregates and H_2_O_2_), contained mitochondrial structures (detected by electron microscopy) and mitochondrial RNA and protein. We propose that in the AD brain, toxicity-causing mitochondrial damage results in the packaging of mitochondrial components for export in EVs and further propose that mt-RNAs in plasma EVs can be diagnostic and prognostic biomarkers for MCI and AD.

## Introduction

Alzheimer’s disease (AD) is the leading age-associated neurodegenerative disease. The disease is associated with numerous structural and functional alterations involving not only neurons but also all other brain cells (Weller and Budson, 2018). Currently, most accepted disease biomarkers relate to the main pathologic hallmarks of the disease: amyloid β (Aβ) plaques (A), MAPT protein (tau) neurofibrillary tangles (T), and neurodegeneration (N). These biomarkers, codified into the A/T/N system, are assessed through invasive and/or expensive tests including cerebrospinal fluid (CSF) sampling and positron emission tomography (PET) scans (Jack et al., 2016). Therefore, extensive efforts are underway to identify robust, specific, sensitive, and easily accessible indicators of AD, particularly at its mild cognitive impairment (MCI) prodromal stage or even preclinically (Sperling et al., 2011; Dubois et al., 2016). In addition to their importance for clinical practice, such biomarkers might inform us about the molecular neuropathology of AD and provide us with novel therapeutic targets.

Extracellular vesicles (EVs) include exosomes (30–150 nm in diameter), which originate from late endosomes and are released from cytoplasmic multivesicular bodies (MVBs) fusing with the plasma membrane, microvesicles (100–1,000 nm in diameter) that originate by budding of the plasma membrane, and apoptotic bodies (reaching up to 5,000 nm in diameter) (Raposo and Stoorvogel, 2013; van Niel et al., 2018). They transport diverse cargo molecules (DNA, RNA, protein, bioactive lipids, etc.) and are believed to have a variety of functions, including disposal of cellular waste and intercellular communication (Hill, 2019). EVs are found in body fluids such as blood, breast milk, amniotic fluid, urine, and saliva (Armstrong and Wildman, 2018) and are produced by all cell types, including neurons, astrocytes, and microglia (Holm et al., 2018; Hill, 2019). The composition, size, and concentration of EVs, and their rate of release vary depending on the cell type and pathophysiological conditions. Accordingly, EV contents from many biological fluids are currently used as diagnostic or prognostic biomarkers. For example, renal EVs detected in blood and urine contain proteins, mRNAs, and microRNAs that are altered with kidney disease and can be utilized as biomarkers (Stahl et al., 2019). Similarly, EVs have emerged as biomarkers in cancer and cardiovascular diseases (Dickhout and Koenen, 2018; Lane et al., 2018).

In the central nervous system (CNS), microglia, oligodendrocytes, neurons, and astrocytes have been found to release EVs (Holm et al., 2018). EVs have been reported to play key roles in the normal CNS (Caruso Bavisotto et al., 2019) and have been implicated in neurodegenerative diseases (Hill, 2019), particularly in the transport of the disease-associated proteins Aβ, tau, and α-synuclein throughout different brain regions (Coleman and Hill, 2015; Budnik et al., 2016; Thompson et al., 2016). Moreover, EVs may travel between the CNS and peripheral circulation and hence can be used as biomarkers for CNS disorders (Shi et al., 2019). In recent years, multiple studies have identified and validated protein cargo of neuronal and astrocytic EVs as biomarkers for AD (Goetzl et al., 2016, 2018; Cha et al., 2019; Kapogiannis et al., 2019b) and other neurological and even psychiatric disorders (Athauda et al., 2019; Bhargava et al., 2019, 2020; Chawla et al., 2019; Kapogiannis et al., 2019a; Mansur et al., 2020). Nevertheless, the rich cargo of EVs provide opportunities for discovering biomarkers that reflect other important aspects of disease pathogenesis.

While the molecular content of EVs can provide critical clues about the mechanisms that govern disease progression, they can also provide a powerful disease signature. The biomarker potential of EV cargo RNAs in AD has been explored mainly for microRNAs in EVs (Cheng et al., 2015; Cha et al., 2019; Lee et al., 2019). In this study, we analyzed the RNAs present in circulating EVs obtained from age-matched individuals who were cognitively normal (healthy controls (HC)), had MCI due to AD or had mild dementia due to AD (five donors per group). RNA-sequencing analysis indicated striking increases in the mitochondrial (mt)-RNA in MCI and AD compared with the HC individuals. This RNA abundance pattern was further validated using reverse transcription (RT) followed by quantitative (q)PCR analysis. As anticipated, the levels of several mt-RNAs including *MT-ND1-6*, *MT-ND4L*, *MT-ATP6*, *MT-ATP8*, *MT-CYTB*, *MT-CO1*, *MT-CO2*, *MT-CO3* mRNAs, and *MT-RNR1* rRNA were elevated in MCI and AD EVs relative to HC EVs, while glyceraldehyde 3-phosphate dehydrogenase (*GAPDH*) mRNA levels remained unchanged. We postulated that toxicity from Aβ and tau aggregates in the AD brain may cause mitochondrial damage in brain cells leading to the export of mitochondrial components to the extracellular space via EVs. To test this hypothesis, cultures derived from astrocytes, microglia, and neurons were incubated with Aβ oligomers, the toxic species in Aβ plaques, or with H_2_O_2_ to mimic the oxidative stress that occurs in the AD environment. EVs released into the respective media following Aβ or H_2_O_2_ treatment showed elevated mt-RNA levels, as well as elevated mitochondrial proteins and remnants of structures resembling mitochondria inside EVs. Together, these findings suggest that the circulating EVs from AD patients contain mitochondrial material, perhaps due to mitochondrial dysfunction that results from exposure to stressful stimuli. Our results further suggest that EV mt-RNAs in plasma may serve as biomarkers of early AD.

## Materials and Methods

### Human Subjects

All participants donated blood as part of their participation in clinical studies at the National Institute on Aging (NIA) approved by the National Institutes of Health Institutional Review Board. All participants provided written informed consent. Procedures for sample collection and processing were identical for all samples. For RNA-sequencing analysis, we used five individuals with mild AD dementia, five with MCI due to AD, and five age- and sex-matched cognitively normal HC (Cohort 1, Table 1). For RT-qPCR analysis, we used another set of five individuals with mild AD dementia, five with MCI due to AD, and five HC individuals (Cohort 2, Table 1).

**TABLE 1:** Demographic, clinical, and biomarker characteristics of human subjects.

	**Normal**	**MCI**	**AD**	***p*-value (AD vs. HC/AD *vs*. MCI)**
**Number of subjects**	**5**	**5**	**5**	
**Cohort 1**				
Age [SD]	76.2 [5.97]	76.0 [7.25]	77.6 [3.65]	NS/NS
Sex (F/M)	2/3	2/3	2/3	NS*
MMSE [SD]		29 [2.07]	23 [2.51]	–/0.020
ADAS-70 [SD]		10 [6.19]	19 [6.40]	–/0.047
CDR-Global [SD]		0.50 [0.00]	0.60 [0.22]	–/NS
CDR-Sob [SD]		1.70 [0.27]	3.60 [1.64]	–/0.052
CSF tau (pg/ml) [SD]		75.60 [15.47]	108.80 [49.09]	–/NS
CSF p181-tau (pg/ml) [SD]		36.40 [11.41]	71.00 [35.77]	–/0.075
CSF Aβ 1-42 (pg/ml) [SD]		155.80 [26.60]	128.60 [39.80]	–/NS
**Cohort 2**				
Age [SD]	75.8	66.40 [3.78]	74.60 [8.29]	NS/0.075
Sex (F/M)	3/2	4/1	3/2	NS/0.003*
MMSE [SD]		25.40 [4.88]	18.60 [6.88]	–/ NS
ADAS-70 [SD]		11.40 [6.23]	22.20 [4.44]	–/0.028
CDR-Global [SD]		0.40 [0.22]	1.20 [0.45]	–/0.005
CDR-Sob [SD]		2.10 [1.39]	6.20 [1.82]	–/0.009
CSF tau (pg/ml) [SD]		63.40 [13.59]	105.00 [66.28]	–/NS
CSF p181-tau (pg/ml) [SD]		56.40 [14.54	59.40 [35.21]	–/NS
CSF Aβ 1–42 (pg/ml) [SD]		184.80 [26.57]	178.40 [22.28]	–/NS

Pairwise comparisons between groups were conducted with t-test for continuous variables and Chi-square for categorical variables. NS corresponds to p > 0.1. * Chi-square.

Individuals with MCI or mild AD dementia for both cohorts were participants in a clinical trial of exenatide in early AD^1^; Mullins et al., 2019); eligibility criteria included > 60 years old, clinical diagnosis of amnestic MCI or probable AD (with mild dementia), clinical dementia rating (CDR) global score of 0.5 or 1, low CSF Aβ42 < 192 pg/ml (using INNO-BIA Alz Bio3 kits), and absence of other neurological disorders or significant neuroimaging abnormalities. For the purpose of this study, we selected baseline samples (i.e., before randomization) of individuals that fulfill criteria for high probability AD based on clinical diagnosis of amnestic MCI or probable AD, low CSF Aβ42, high CSF total tau, and/or p181-tau (Albert et al., 2011; McKhann et al., 2011). Moreover, upon review of individual cases, individuals used for this study qualify as amyloid+/tau+/neurodegeneration+ according to the A/T/N framework (Jack et al., 2016). Normal controls were participants at the Baltimore Longitudinal Study on Aging conducted at the NIA who have remained cognitively normal for the course of their participation; they have Blessed Information Memory Concentration Test score < 4, and CDR = 0. EVs isolated from each individual subject were processed and analyzed separately.

### RNA Sequencing Analysis From EVs in Human Plasma

Plasma was isolated from five HC, five MCI, and five AD donors (Cohort 1). EV RNA was extracted from 4 ml of thawed human plasma using exoRNeasy Serum/Plasma Maxi kits (QIAGEN) and cDNA was prepared and amplified using the Ovation RNA-Seq System V2 (NuGEN) kit. The amplified cDNA was fragmented using a Bioruptor (Diagenode), adaptors were ligated to cDNA using TruSeq ChIP Sample Preparation kit (Illumina, San Diego, CA, United States), the DNA fragments were size-selected (300–350 bp) after electrophoresis on a 2.5% agarose gel, the selected DNA was subjected to 17 cycles of PCR amplification, and library quality was determined using a Bioanalyzer 2100. The final libraries were subjected to paired-end sequencing (110 bases) using an Illumina HiSeq 2500 sequencer. FASTQ files were extracted using bcl2fastq v2.18.0.12, trimmed for adapter sequences using Cutadapt v1.18, and aligned to human genome hg19 Ensembl v82 using STAR software v2.4.0j. FeatureCounts was used to create gene counts from 15 samples. Differential expression analysis was carried out using edgeR library in R. Data are available at GSE153881.

### Reverse Transcription and Real-Time Quantitative (q)PCR Analysis From EVs

For validation studies, plasma was collected from a separate set of individuals (five HC, five MCI, and five AD). EVs isolated from individual subjects were not pooled, and their effects were assessed and analyzed separately to respect their biological variability.

For RT-qPCR validation of RNA in EVs, 1 ml each of thawed human plasma from five individuals per group (HC, MCI, and AD) was filtered using 0.8 μm Millex-AA syringe filters (Millipore) to eliminate larger particles including apoptotic bodies. EV RNA was then extracted using exoRNeasy Serum/Plasma Kit (QIAGEN) and used for cDNA synthesis using random hexamers and reverse transcriptase (Invitrogen). Gene-specific primers were then used for real-time quantitative (q)PCR amplification by employing SYBR Green master mix (Kapa Biosystems) and a QuantStudio 5 thermal cycler (Thermo Fisher Scientific). PCR primer pairs (each forward and reverse) were as follows:

CCCACTTCTTACCACAAGGC and GTAGGTGGCCTGCA GTAATG for *MT-ATP6* mRNA, ATGCCCCAACTAAATACT and TTGTGGGGGCAATGAATG for *MT-ATP8* mRNA, ACC CTAGACCAAACCTACGC and TAGGCCGAGAAAGTGTTG TG for *MT-CO1* mRNA, ACAGATGCAATTCCCGGACG and GGCATGAAACTGTGGTTTGC for *MT-CO2* mRNA, ACTTCCACTCCATAACGCTC and TGGCCTTGGTATGTGC TTTC for *MT-CO3* mRNA. CTCCCGTGAGGCCAAATATC and GAATCGTGTGAGGGTGGGAC for *MT-CYTB* mRNA, TG CACCACCAACTGCTTAGC and GGCATGGACTGTGGTCA TGAG for *GAPDH* mRNA, TGGCCAACCTCCTACTCCTC and ATGGCGTCAGCGAAGGGTTG for *MT-ND1* mRNA, ACTGC GCTAAGCTCGCACTG and ATTATGGATGCGGTTGCTTG for *MT-ND2* mRNA, CTACCATGAGCCCTACAAAC and ACT CATAGGCCAGACTTAGG for *MT-ND3* mRNA, ACAAGCTC CATCTGCCTACG and TTATGAGAATGACTGCGCCG for *MT-ND4* mRNA, TATCGCTCACACCTCATATC and AGGCG GCAAAGACTAGTATG for *MT-ND4L* mRNA, GGTTTCA TCCTCGCCTTAGC and ACCTAATTGGGCTGATTTGC for *MT-ND5* mRNA, ATTGGTGCTGTGGGTGAAAG and GGATCCTCCCGAATCAACCC for *MT-ND6* mRNA, and CT GCTCGCCAGAACACTACG and TGAGCAAGAGGTGGTGA GGT for *MT-RNR1* rRNA.

For RT-qPCR quantification of RNAs present in EVs prepared from mouse primary neurons, the following primer pairs were used (each forward and reverse):

GGATGCAGGGATGATGTTCT and GGGTGTGAACCACG AGAAAT for *Gapdh* mRNA, CATTAGCAGTCCGGCTTACA and GTAGCTGTTGGTGGGCTAAT for *mt-Atp6* mRNA, TGTATGAGCCCACCACATATTC and CACCGGTAGGAAT TGCGATAA for *mt-Co1* mRNA, AGGAGACCCAGACAAC TACA and TGAGCGTAGAATGGCGTATG for *mt-Cytb* mRNA, CCATTTGCAGACGCCATAAA and GAGTGATAGGGTAGGT GCAATAA for *mt-Nd1* mRNA, TGGTTGTCTTGGGTTAGCA TTA and AACGATCCACCAAACCCTAAA for *mt-Nd6* mRNA, and CAGCCTATATACCGCCATCTTC and TTGGCTACACCT TGACCTAAC for *mt-Rnr1* rRNA.

### Cell Culture and Treatment Conditions

Immortalized human microglia HMC3 cells (ATCC CRL−3304), astrocytoma 1321N1 cells (Sigma-Aldrich; derived from human brain astrocytoma; Macintyre et al., 1972), and neuroblastoma SK-N-BE(2)-M17 (M17) (ATCC CRL-2267) cells were cultured in Eagle’s minimum essential medium (EMEM) or Dulbecco’s modified Eagle’s medium (DMEM) supplemented with 10% fetal bovine serum (FBS, Gibco) and 1% antibiotics and antimycotics (Gibco).

For primary cultures of embryonic cortical neurons, timed-pregnant mice were obtained from The Jackson Laboratory. Cultures were prepared from embryonic day (E) 14.5 cerebral tissues as described (Zhao et al., 2019). Briefly, pregnant mice were killed by fast cervical dislocation, embryos and embryo brains were removed, and the cerebral hemisphere was dissected in sterile Hank’s balanced saline solution (HBSS). The collected brain tissues were incubated in 0.25% trypsin-EDTA for 30 min at 37°C and then transferred to DMEM containing 10% fetal bovine serum (DMEM+). The tissues were transferred to neurobasal (NB) medium containing B27 supplements, 2 mM L-glutamine, antibiotics and antimycotics (Gibco), and 1 mM HEPES and dissociated by trituration using a fire-polished Pasteur pipet. The dissociated cells were seeded into polyethyleneimine-coated plastic culture dishes at a density of 60,000 cells/cm^2^ and cultured in the same B27-containing NB medium.

Before the collection of EVs from conditioned culture media, cells were incubated for 3 days in media containing exosome-depleted FBS (Gibco). Cell lines HMC3 and 1321N1 were treated for 3 days with either 50 or 100 μM hydrogen peroxide (H_2_O_2_; Sigma-Aldrich). H_2_O_2_ was diluted from a 10 mM stock solution immediately before use. Primary neuronal cultures at day 3 of culture *in vitro* (DIV 3) were treated with either 5 μM H_2_O_2_ for three additional days and collected at DIV 6. Aβ_1__–__42_ (Aβ42, Bachem) was dissolved in double-deionized water at 200 μM of Aβ42 by incubation at 37°C and further diluted with conditioned media for 24 h to promote peptide self-aggregation to obtain the concentration of 1 or 5 μM of oligomeric Aβ.

### EV Isolation From Cell Culture Media

For human cell lines HMC3, M17, and 1321N1, cells were grown in 150-mm dishes with DMEM or EMEM with 10% EV-depleted FBS until cells reached approximately 90% confluency (3 days). Primary neuronal cultures were cultured in 60-mm dishes for 3 days (DIV 3), whereupon they were changed to NB media without FBS; 3 days later (at DIV 6), the conditioned media was collected for EV isolation. Cell viability was assessed using ViaStain AO/PI Staining Solutions (Nexcelom). Medium from cultures with > 85% viability was used for EV RNA isolation, which was performed using exoRNeasy Serum/Plasma Maxi kits (QIAGEN) following the manufacturer’s protocol. The intact EVs were isolated from the media following the protocol developed by Jeppesen et al. (2019). Briefly, media was centrifuged at 500 × *g* for 10 min at room temperature, and further centrifuged at 2000 × *g* for 20 min to remove debris and apoptotic bodies. From this supernatant, samples were pelleted by centrifugation at 15,000 × *g* for 40 min and EVs by centrifugation at 100,000 × *g* for 16 h at 4°C. The EVs were resuspended in 4 ml of filtered PBS (0.22-μm filter, Millipore) followed by additional ultracentrifugation at 55,000 rpm for 1 h using a TLA-110 Rotor (Beckman Coulter, Fullerton, CA) to wash the sample. EVs were centrifuged 55,000 rpm at 4°C to pellet for RNA isolation (Trizol, Invitrogen), protein extraction, and electron microscopy analysis.

### Protein Analysis

Proteins were extracted from whole-cell lysates or EVs using RIPA buffer (50 mM Tris-HCl (pH 7.2), 150 mM NaCl, 1% NP40, 0.1% SDS, 0.5% DOC, 1 mM PMSF, 25 mM MgCl_2_) supplemented with a Halt^TM^ Protease and Phosphatase Inhibitor Cocktail (Thermo Scientific). Following size fractionation using SDS-containing polyacrylamide gels, samples were transferred onto nitrocellulose membranes (Bio-Rad Laboratories). Primary antibodies recognizing TOMM20, COX IV, OPA1, and GAPDH (Cell Signaling Technology), CD81 (Abcam) and CD63 (BD Biosciences) were used. After incubation with appropriate secondary antibodies, protein signals were developed using enhanced chemiluminescence (ECL), and digitized images were captured using Kwik Quant Imager (Kindle Biosciences).

### Transmission Electron Microscopy

Samples were adhered to Poly-L-Lysine-coated coverslips for 5 min, then fixed in 1% glutaraldehyde 80 mM phosphate buffer (Sorenson’s) containing 5 mM MgCl_2_ at pH 7.4. One hour later, samples were placed in the same fixative with the pH shifted to 8.5 and fixed for 1 h at room temperature. Coverslips were rinsed in buffer containing sucrose then postfixed in potassium ferrocyanide-reduced osmium tetroxide for 1 h on ice. After phosphate and maleate buffer rinses, samples were stained *en bloc* in 2% uranyl acetate in maleate buffer for 1 h at 4°C. After a series of ethanol dehydrations (30–100%), samples were infiltrated with Eponate 12 (Polysciences) resin, embedded, and cured at 60°C for 48 h.

Coverslips mounted to inverted beam capsules were gently removed after soaking in liquid nitrogen for 10 min. Blocks were then trimmed and sectioned on a Riechert Ultracut E microtome with a Diatome Diamond knife (45°). Sections (70 nm) were picked up on formvar-coated 1 × 2 mm copper slot grids and stained with methanolic uranyl acetate followed by lead citrate. Grids were viewed on a Hitachi H 7600 TEM operating at 80 kV and digital images captured with an XR50 5 megapixel CCD camera from Advanced Microscopy Techniques Corp.

## Results

### Increased Abundance of mt-RNAs in EVs From MCI and AD

**FIGURE 1:**
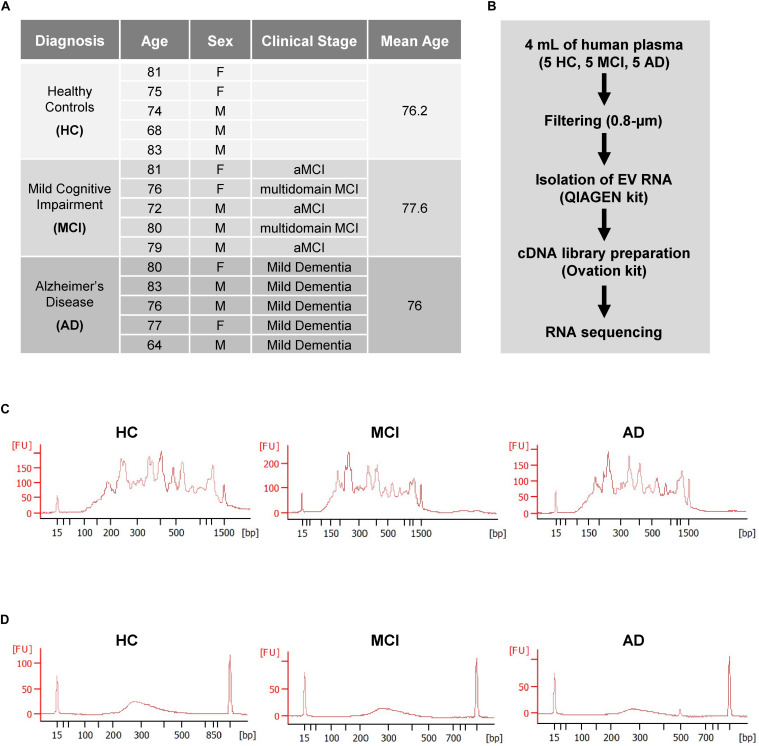
Preparation of extracellular vesicles (EVs) for RNA sequencing (RNA-seq) analysis. **(A)** Subject characteristics of individual human blood donors for RNA-seq analysis. Five samples were collected in each group (HC, healthy donors; MCI, patients with mild cognitive impairment due to AD; AD, patients with mild dementia due to Alzheimer’s disease). Mean Age is 76.2 for healthy donors, 77.6 for MCI, and 76 for AD. **(B)** Workflow of EV isolation, RNA extraction, and RNA-seq analysis. **(C,D)** Bioanalyzer chronographs of cDNA amplified by ovation kit **(C)** and cDNA libraries **(D)**. Data in **(C**,**D)** are representative of individuals in each of the 3 groups.

Each group of HC, MCI, and AD individuals included three males (M) and two females (F) with mean ages of 76.2, 77.6, and 76 years, respectively (Cohort 1, section “Materials and Methods”; Figure 1A). As outlined in the workflow (Figure 1B), donor plasma samples were used to isolate total RNA from circulating EVs, which was then used to prepare cDNA libraries using a NuGEN kit (section “Materials and Methods”). Representative data of successful amplification using the ovation kit and reduction of distinct ribosomal RNA peaks are shown (Figures 1C,D). RNA sequencing (RNA-seq) analysis was performed as described (section “Materials and Methods”) with 110 bp paired-end directional reads.

**FIGURE 2:**
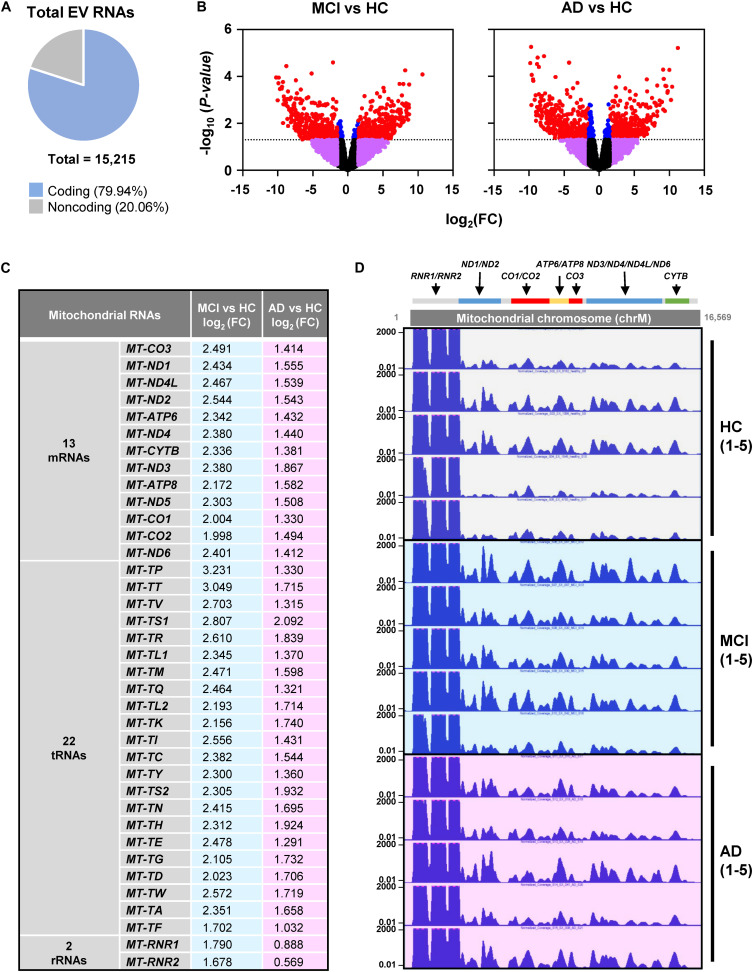
RNA-sequencing (RNA-seq) analysis. **(A)** Pie chart of total transcripts in EVs (for breakdown of noncoding RNA biotypes, see Supplementary Figure S1). **(B)** Volcano plot of significantly changed transcripts, MCI vs. HC and AD vs. HC. **(C)** List of mitochondrial RNAs that are found in EVs using RNA-seq analysis: 13 mitochondrial mRNAs, 22 mitochondrial tRNAs (transfer RNAs), and 2 mitochondrial rRNAs (ribosomal RNAs). Changes in abundance were quantified as log_2_ fold change (FC) differences in MCI vs. HC and AD vs. HC. **(D)** UCSC genome browser of mitochondrial chromosome (Chr M) from 5 HC, 5 MCI, and 5 AD persons. Mitochondrial genes were indicated above the Chr M schematic.

RNA-seq analysis revealed a total of 15,215 transcripts in all EVs. The majority of RNAs (∼80%) corresponded to coding transcripts (mRNAs), while non-coding RNAs (e.g., pseudogene RNAs, lincRNAs, mt-tRNAs, antisense RNAs, etc.) comprised ∼20% of the total (Figure 2A and Supplementary Figure S1A). Comparisons of the EV RNAs from MCI relative to HC and from AD relative to HC individuals revealed many differentially abundant RNAs in these samples, as shown in the volcano plots (Figure 2B). Strikingly, mt-RNAs were among the most abundant RNAs in all samples; among mt-RNAs, we found 13 mRNAs, 22 tRNAs, and 2 rRNAs, all upregulated in MCI, and most of these are also upregulated in AD (Figure 2C). Non-coding RNAs transcribed in the nucleus but capable of residing in mitochondria in relatively low levels (e.g., *RMRP*, *RPPH1*, *TERC*) were undetectable (not shown). The abundance of reads of mt-RNAs found in EVs (from HC, MCI, and AD donors), along the mitochondrial chromosome were visualized in the UCSC Genome Browser (Figure 2D). As observed, the mitochondrial RNA reads in EVs from HC individuals (gray) were lower than the mitochondrial RNA reads from MCI (blue) or AD (pink). These findings indicate that EVs from persons with MCI and AD have higher levels of mitochondrial RNAs than EVs from healthy persons.

### Validation of mt-RNAs

**FIGURE 3:**
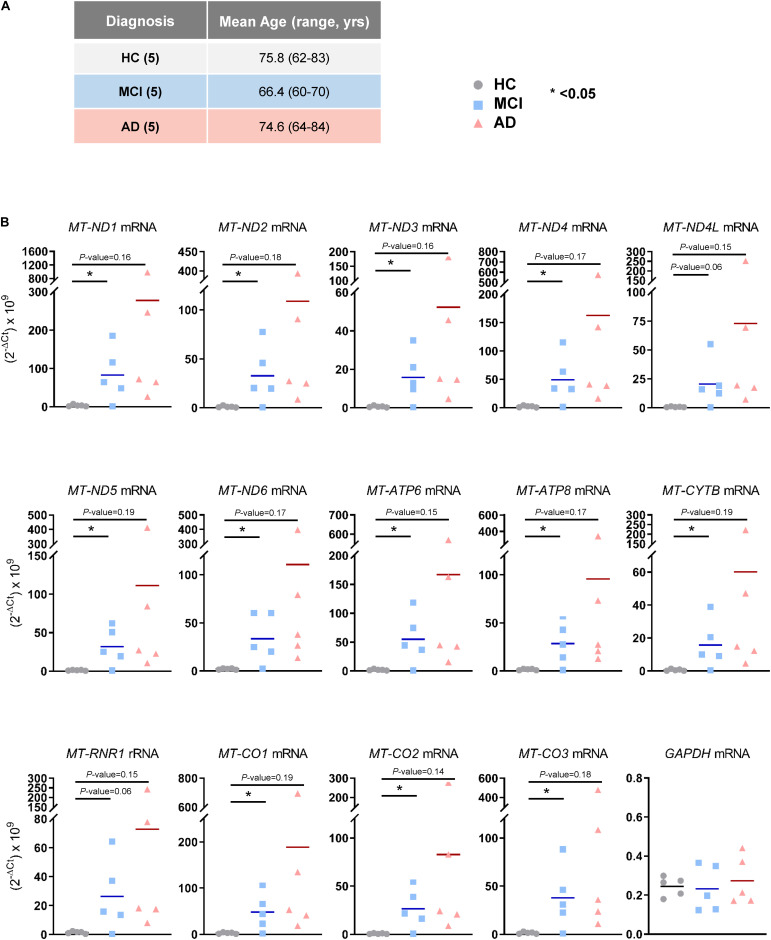
Validation of mt-RNAs in EVs by RT-qPCR analysis. **(A)** Subject characteristics of additional individual human blood donors for RNA-sequencing validation. Five samples were collected in each group as in Figure 1A. Mean age is 75.8 years for HC, 66.4 years for MCI, and 74.6 years for AD. **(B)** RT-qPCR analysis of human mt-RNAs (*MT-ND1*, *MT-ND2*, *MT-ND3*, *MT-ND4*, *MT-ND4L*, *MT-ND5*, *MT-ND6*, *MT-ATP6*, *MT-ATP8*, *MT-CYTB*, *MT-CO1*, *MT-CO2*, *MT-CO3* mRNAs, and *MT-RNR1* rRNA) and *GAPDH* mRNA. Data represent all individuals of each group, 5 HC, 5 MCI, and 5 AD.

The observation that mt-RNAs were widely elevated in EVs from MCI and AD individuals, as determined from RNA-seq analysis, prompted us to validate these changes using RT-qPCR analysis in a new cohort. We obtained plasma samples from an additional five individuals per group (HC, MCI, and AD in Cohort 2, section “Materials and Methods”; Figure 3A), isolated EVs, extracted RNAs, and performed RT-qPCR analysis using specific primer pairs. As shown in Figure 3B, all transcripts tested (*MT-ND1, MT-ND2, MT-ND3, MT-ND4, MT-ND4L, MT-ND5, MT-ND6, MT-ATP6, MT-ATP8, MT-CYTB, MT-CO1, MT-CO2*, and *MT-CO3* mRNAs, and *MT-RNR1* rRNA) were significantly more abundant in EV RNAs obtained from individuals with MCI due to AD (but not from individuals with mild AD dementia) than in EV RNA from HC individuals. Measurement of *GAPDH* mRNA, which encodes the housekeeping protein GAPDH, showed that the levels of this cytoplasmic mRNA were unchanged among these groups. In summary, in EVs from patients with MCI due to AD, the levels of mt-RNAs are robustly and significantly more abundant.

### Brain Cell Types Release EV-Containing mt-RNA Upon Stress

Given the strong rise in mt-RNAs in EVs from MCI and AD individuals, we hypothesized that this elevation might result from the toxic environment in the AD brain from the accumulation of Aβ and other neurotoxic aggregates causing oxidative damage. Of course, EVs enriched in mt-RNAs may also originate from other tissues and organs of MCI and AD individuals, since we were unable to establish the tissue of origin (see section “Discussion”). Thus, we set out to test the hypothesis that brain cells present in the vicinity of neurotoxic stimuli (Aβ and oxidants) might participate in the release of EVs bearing mt-RNAs, and that these EVs would then cross the blood-brain barrier (BBB) and appear in plasma. To study this possibility, we employed cultured human astrocytoma 1321N1 cells, microglia (HMC3) cells, and neuroblastoma M17 [SK-N-BE(2)-M17] cells, as well as mouse primary neurons, and exposed them to different doses of oligomeric Aβ (specifically Aβ_1__–__42_, “Aβ42”) and the oxidant hydrogen peroxide (H_2_O_2_) continuously for 3 days, whereupon we isolated EVs from the conditioned media and extracted RNA for RT-qPCR analysis of mt-RNAs. Control EVs were prepared after incubating cells with filtered PBS instead of Aβ42 oligomers or H_2_O_2_. In control reactions, the presence of abundant transcripts often found in EVs (*e.g.*, *GAPDH* and *ACTB* mRNAs) and the absence of transcripts often undetectable in EVs (*e.g.*, *CDKN1A* and *CDKN2A* mRNAs) were confirmed by RT-qPCR analysis (Supplementary Figure S2A); mitochondrial DNA was also undetectable (not shown).

**FIGURE 4:**
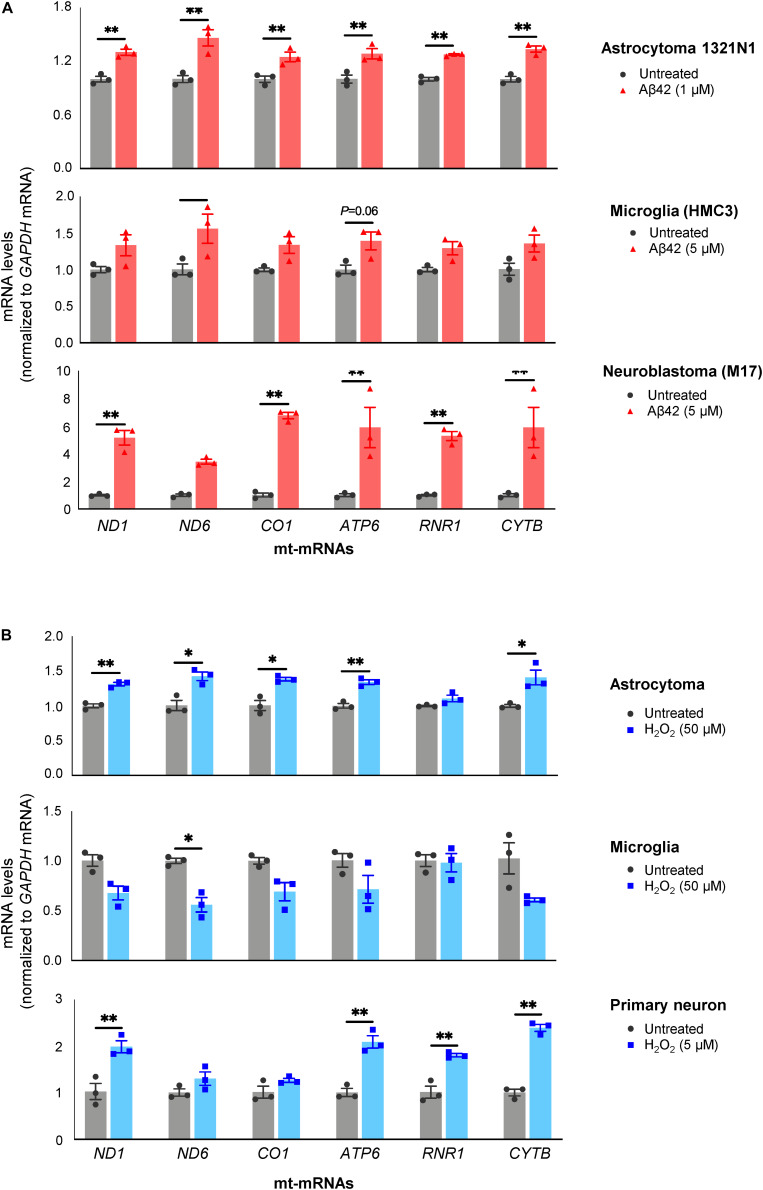
Treatment with Aβ42 and H_2_O_2_ increases mt-RNAs in EVs. **(A)** RT-qPCR analysis of mt-RNAs in EVs obtained from human astrocytoma, microglia, and neuroblastoma cells after treatments with Aβ42 oligomers, as indicated (section “Materials and Methods”). **(B)** RT-qPCR analysis of mt-RNAs in EVs obtained from astrocytoma, microglia, or primary mouse neurons after treatments with H_2_O_2_ as indicated (section “Materials and Methods”). Data are normalized to *GAPDH* mRNA values and represent the means ± SEM from three independent experiments. *P < 0.05, **P < 0.01.

As shown in Figure 4A, all three Aβ42-treated populations released EVs containing more mt-RNAs than untreated cells. Both H1321N1 cells (treated with 1 μM Aβ42) and M17 cells (treated with 5 μM Aβ42) showed significantly higher levels of *ND1*, *ND6*, *CO1*, *ATP6*, and *CYTB* mRNAs, and *RNR1* rRNA upon Aβ treatment compared with cells treated with vehicle alone. Microglial cells (HMC3) showed a similar trend but to a lesser extent (Figure 4A). Similarly, treatment with H_2_O_2_ also increased levels of mt-RNAs in EVs released by astrocytoma 1321N1 cells (Figure 4B) and from neuroblastoma M17 cells (not shown), although not from microglia (Figure 4B). We also observed that H_2_O_2_ treatment increased mt-RNA levels in EVs from mouse primary neurons (Figure 4B). Together, these findings support the notion that stressors such as Aβ42 and the oxidant H_2_O_2_ trigger the release of EVs containing high levels of mt-RNAs.

### Other Mitochondrial Contents in EVs in Response to Stress

The above findings support the notion that in the neurotoxic environment of the AD brain, astrocytes, microglia, and neurons might release more EVs that contain mt-RNA, possibly because mitochondrial fragments are released in such EVs. To further test this hypothesis, we investigated whether EVs released in these cultured models (Aβ42- and H_2_O_2_-treated brain cells) contained mitochondria or mitochondria fragments as seen using transmission electron microscopy (TEM) analysis; control EVs were again collected after incubating cells with filtered PBS instead of Aβ42 oligomers or H_2_O_2_. For this analysis, we focused on 1321N1 astrocytoma cells treated with toxic agents. Following Aβ42 treatment and collection and preparation of EVs, TEM images did not reveal intact mitochondria in the EV preparations but did detect many membranous remnants inside EVs of different sizes (arrows), which could be mitochondria or other organelles.

**FIGURE 5:**
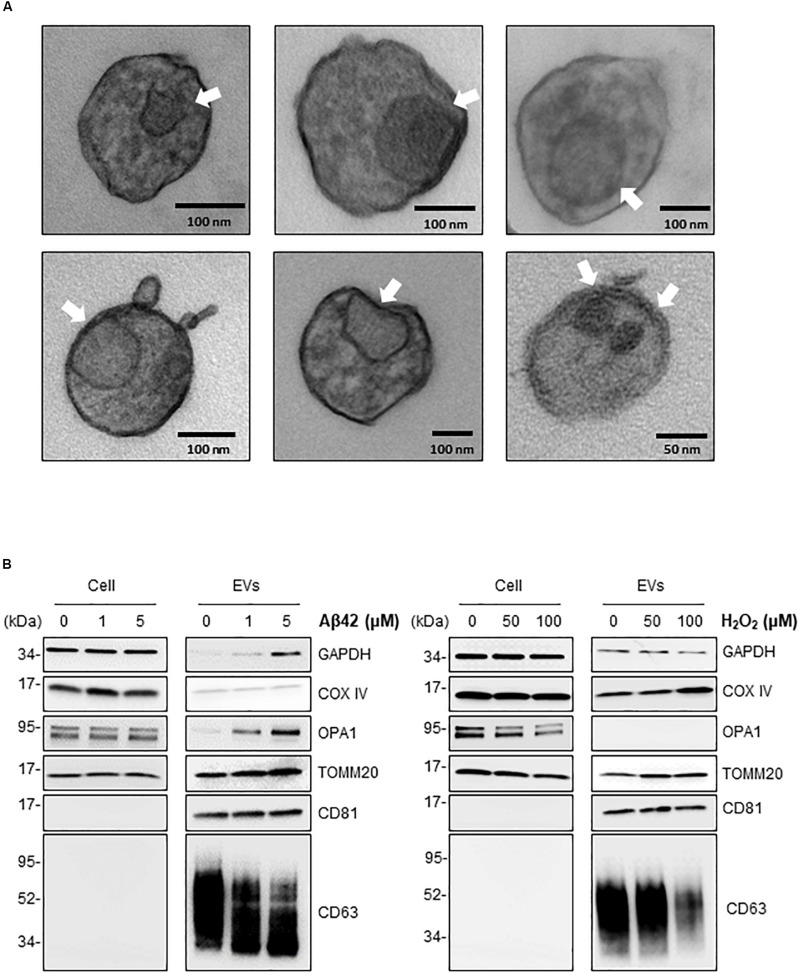
Treatment of astrocytoma with Aβ42 and H_2_O_2_ increases the presence of inclusions and mitochondrial proteins in EVs. **(A)** EVs were isolated from astrocytoma cells treated with 1 μM Aβ42 for 3 days. Representative transmission electron microscopy (TEM) images showing inclusions of structures (arrows) consistent with mitochondria fragments. Black bars, size scale. **(B)** Western blot analysis of COXIV, OPA1, TOMM20, GAPDH, CD81, and CD63 using whole-cell lysates (‘Cell’) and EVs obtained from astrocytes after the indicated treatments.

**FIGURE 6:**
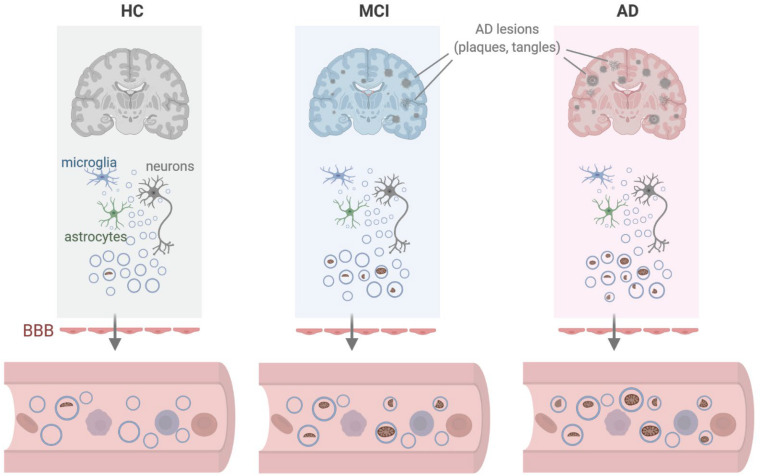
Model. We propose that in the vicinity of lesions (plaques, tangles) found in persons with mild cognitive impairment (MCI) or Alzheimer’s disease (AD), local damage to brain tissues can lead to the release of EVs containing dysfunctional or damaged mitochondria from cells including microglia, astrocytes, and neurons. As these EVs cross the blood-brain barrier (BBB), the mitochondrial contents (including mitochondrial RNA) present as EV cargo can be measured in peripheral blood. Created in BioRender.

To ascertain whether the structures identified by TEM might include mitochondrial material, we performed Western blot analysis to assess the levels of mitochondrial proteins in EVs under stress conditions. Consistent with the mt-RNA analysis, we also detected higher levels of mitochondrial proteins in EVs released by 1321N1 astrocytoma cells treated with Aβ42 and H_2_O_2_ relative to untreated cells (Figure 5B). As shown, the levels of TOMM20 (translocase of outer mitochondrial membrane 20) increased in astrocytoma-released EVs upon Aβ42 and H_2_O_2_ treatments; the levels of TOMM20 in whole-cell lysates were not significantly altered by Aβ42 treatment but decreased following H_2_O_2_ treatment. The levels of mitochondrial cytochrome *c* oxidase subunit IV (COX IV) increased upon H_2_O_2_ treatment but not by Aβ42 treatment (Figure 5B), while the levels of the mitochondrial protein optic atrophy protein 1 (OPA1) increased in EVs from Aβ42-treated cells but were undetectable in EVs from H_2_O_2_-treated cells. CD81, a canonical EV marker used as positive control, was only detected in EVs and not in whole-cell lysates (‘Cells’, Figure 5B). GAPDH signals were included as control for loading of whole-cell lysates; other loading assessments are shown in Supplementary Figures S2B,C. Together, these data support the possibility that mt-RNAs and mitochondrial proteins found in EVs may be released as mitochondrial fragments from damaged cells (Figure 6). The data further suggest that the increased levels of mt-RNAs recovered from plasma EVs of MCI and AD individuals could originate (at least in part) from mitochondrial fragments of brain cells released into EVs in regions of neurotoxicity.

## Discussion

Here, we investigated the RNA content of circulating EVs isolated from the blood of HC, MCI, and AD individuals and discovered a striking enrichment in mitochondrial RNAs in the MCI and AD groups (Figure 2). This finding suggests that the levels of mt-RNAs in circulating EVs could be developed into a novel diagnostic biomarker for early AD. Using a different cohort of plasma donors, we carried out RT-qPCR analysis to validate the RNA-seq differences between HC and MCI (although not the difference from HC to AD due to high variability), further suggesting that EV-associated mt-RNAs may be used as AD biomarkers, especially for earlier stages of the disease (Figure 3).

AD is the result of multiple overlapping pathogenic processes, including mitochondrial dysfunction. AD lesions, particularly Aβ aggregates, trigger the production of reactive oxygen species (ROS) and damaged mitochondria, which in turn causes oxidative injury (Shelat et al., 2008), and at the same time, mitochondrial dysfunction has been documented extensively in AD brain (Beal, 1998 and Beal, 2005). The discovery that circulating EVs of AD individuals contain markedly higher levels of mt-RNAs raises a pressing question: what cells generate these EVs rich in mt-RNAs? The use of cell type-specific markers on the surface of EVs to isolate EV sub-populations could help resolve this question, but, since immunocapture methodologies result in > 10-fold lower concentrations of EVs (Mustapic et al., 2017) and correspondingly lower RNA levels, it was not feasible to use them for this study, which relied on RNA-seq analysis. Our effort to immunocapture EVs enriched for neuronal origin using anti-L1CAM antibodies (Suire et al., 2017) resulted in a yield of EVs that was too low to allow amplification of even the most abundant RNAs; similar limitations were anticipated with the detection of the astrocyte-specific EV marker GLAST [astrocyte cell surface antigen-1 (ACSA-1)] (not shown). Accordingly, it is important to recognize that the mt-RNA-bearing EVs identified here might also originate in other tissues and organs outside of the brain.

As an alternative approach, we sought to obtain preliminary answers through experiments using cultured cells from brain, as we posited that brain cells might be the source of EVs with elevated mt-RNAs in AD and MCI. To model the neurotoxic niche in the vicinity of Aβ plaques in culture, we treated different brain cells with toxic Aβ42 oligomers or oxidants, and quantified the RNA content of the EVs released. As documented in Figure 4, human cells derived from microglia and astrocytes, as well as neurons from mouse (primary neurons) and human (neuroblastoma cells) exposed to Aβ42 oligomers or H_2_O_2_ for 3 days released EVs that were enriched in mt-RNAs. These results support the notion that microglia, astrocytes, and neurons from the neurotoxic niche may contribute to the population of circulating EVs bearing high levels of mt-RNAs. Although the magnitude of mt-RNA enrichment in EVs from Aβ42 or H_2_O_2_-treated cells was far lower than that observed in plasma of AD individuals, the time scales in each case are very different, as cultured cells were only exposed for 3 days while the brain cells of individuals with AD are exposed to neurotoxic stimuli over decades and their EVs potentially reach a high steady concentration in plasma over months or years. It is also certainly possible that other cell types in the body of individuals with AD may release these EVs instead or in addition to brain cells. Further technical development to capture specific cell EVs with higher yield may further reveal the source of mt-RNA-enriched circulating EVs. In addition to mt-RNAs, EVs released from glial and neuronal cells challenged with Aβ42 or H_2_O_2_ contained membranous remnants consistent with mitochondrial fragments (Figure 5) as well as mitochondrial proteins which also warrant testing as biomarkers. The specific size and composition of the mt-RNA-bearing EVs remain to be studied in molecular detail. However, these EVs do not appear to include apoptotic bodies, as we did not observe apoptotic markers (cleaved caspase 3 or cleaved PARP) in the cell populations and the diameter of the EVs (<600 nm) was smaller than the typical diameter (< 1,000 nm) of apoptotic bodies (Supplementary Figure S3). It remains to be determined if EVs in AD plasma samples contain mitochondrial proteins or fragments similar to those found in EVs in culture.

Why might neurotoxic stimuli trigger the release of mitochondrial content in cultured cells? We postulated that cells might remove damaged mitochondria in order to prevent further oxidative injury. The removal of damaged mitochondria can be achieved internally by the cell through mitophagy, a form of autophagy specialized in breaking down dysfunctional mitochondria (Youle and Narendra, 2011; Yin et al., 2016; Montava-Garriga and Ganley, 2020; Wang et al., 2020). Such elimination of dysfunctional mitochondria through mitophagy and the mitochondrial-lysosomal axis become aberrant during aging (Picca et al., 2019a). Therefore, in a chronic disease developing against the background of aging, such as AD, perhaps the burden of damage is such that removal of dysfunctional mitochondria from the cell by encapsulating them in EVs may be chosen to accelerate this clearance.

EVs have also been proposed to function in intercellular communication and transport cargo from one cell or tissue to another, possibly spanning long distances. Whether EVs from AD individuals may have a signaling role is unknown. However, it is unlikely that the mt-RNAs being transported in EVs can function in protein translation, as the mt-RNA in our EVs are fragmented (Figures 1C,D). Nonetheless, small RNAs, peptides, bioactive lipids, and small molecules present in EVs from MCI and AD plasma might function as signaling factors in cell-to-cell communication (El Andaloussi et al., 2013; Thompson et al., 2016), but their nature and function have not been comprehensively studied at present.

Other pathologies have also been associated with the presence of mitochondrial contents in EVs. For example, sera from children with autism spectrum disorder contain a significant amount of mt-DNA compared with the normotypic controls (Tsilioni and Theoharides, 2018), astrocytes release mitochondrial particles after stroke (Hayakawa et al., 2016), and EVs containing mitochondria components were proposed as biomarkers in PD (Picca et al., 2019b). In addition, mitochondrial membrane proteins were also higher in melanoma-derived EVs compared with control EVs (Jang et al., 2019) and in chemoresistant breast cancer (Sansone et al., 2017). Adding mt-RNA and other RNAs to protein biomarkers may further increase the diagnostic performance of EV biomarkers in AD. As the presence of mitochondrial material in EVs is increasingly being associated with various diseases, it will be important to comprehensively study mitochondrial molecules as biomarkers of disease and aging.

## Data Availability Statement

The datasets presented in this study can be found in online repositories. The names of the repository/repositories and accession number(s) can be found below: https://www.ncbi.nlm.nih.gov/geo/, GSE153881.

## Ethics Statement

The studies involving human participants were reviewed and approved by the Institutional Review Board NIA NIH. The patients/participants provided their written informed consent to participate in this study.

## Author Contributions

KK, QM, DK, and MG conceived the manuscript. KK QM, OP, MM, AC, EE, RM, WW, JN, and MD performed the experiments. GK and SD performed the bioinformatic analysis. LC and KA contributed intellectually. KK, QM, DK, and MG wrote the manuscript. All authors contributed to the article and approved the submitted version.

## Conflict of Interest

The authors declare that the research was conducted in the absence of any commercial or financial relationships that could be construed as a potential conflict of interest.

## Footnotes

1 https://clinicaltrials.gov/ct2/show/NCT01255163
