# Supplementary material for: Mitochondrial RNA in Alzheimer’s Disease Circulating Extracellular Vesicles
Source: Front Cell Dev Biol. 2020 Nov 16;8:581882. doi: 10.3389/fcell.2020.581882 (PMC7701247; doi:10.3389/fcell.2020.581882)

Mitochondrial RNA in Alzheimer's Disease Circulating Extracellular Vesicles

Kyoung Mi Kim[1,3,*], Qiong Meng[1,*], Olivia Perez de Acha[1], Maja Mustapic[2], Aiwu Cheng[1], Erden Eren[2], Gautam Kundu[1], Yulan Piao[1], Rachel Munk[1], William H. Wood III[1], Supriyo De[1], Ji Heon Noh[4], Michael Delannoy[5], Lesley Cheng[6], Kotb Abdelmohsen[1], Dimitrios Kapogiannis[2,**], and Myriam Gorospe[1,**]

[1]Laboratory of Genetics and Genomics and [2]Laboratory of Clinical Investigation, National Institute on Aging Intramural Research Program, National Institutes of Health, Baltimore, MD, USA; [3]Department of Biological Sciences and [4]Department of Biochemistry, Chungnam National University, Daejeon, Republic of Korea; [5]Department of Cell Biology and Imaging Facility, Johns Hopkins University School of Medicine, Baltimore, MD, USA; [6]Department of Biochemistry and Genetics, School of Molecular Science, La Trobe University, Mellburne, Australia.

*Co-first authors

**Correspondence:
National Institute on Aging IRP, NIH
251 Bayview Blvd, Baltimore, MD 21224, USA
kapogiannisd@mail.nih.gov; +1-410-350-3953
myriam-gorospe@nih.gov; +1-410-454-8412

Running title: mt-RNAs in AD EVs

SUPPLEMENTAL FIGURE LEGENDS

Figure S1. Additional information on RNA-seq analysis of plasma EVs from AD and MCI individuals. (A) Percentage of RNA reads mapping to different types of transcripts in EVs from human plasma samples. **(B)** Heatmap of the 37 mt-RNAs (MCI vs. HC, AD vs. HC). Scale indicates Log_2(Fold change).

Figure S2. Additional analysis of protein contents in EVs from cultured cells following neurotoxic damage. (A) RT-qPCR analysis of RNA present in EVs from astrocytoma cells treated as indicated to quantify the levels of several control transcripts, including *GAPDH* and *ACTB* mRNAs, highly abundant in EVs, and *CDKN2A* and *CDKN1A* mRNAs, in low abundance in EVs. Data represent the means ± S.E.M. from three independent experiments. **(B,C)** Ponceau S staining of the Western blots shown in Figure 5 using astrocytoma cell lysates and secreted EVs following treatment with Aβ42 (B) or H_2O_2 (C).

Figure S3. Additional analysis of protein contents in cultured cells and the Size characterization of EVs by Nanoparticle Tracking Analysis (NTA). (A) Western blot analysis of the levels of apoptotic markers cleaved PARP and cleaved caspase-3 as well as loading control GAPDH in whole-cell lysates obtained from astrocytes after the indicated treatments with Aβ42 or H_2O_2. Western blot analysis of whole-cell lysates from microglia cells treated with etoposide for 18 h were included as positive controls for cleaved caspase 3 and cleaved PARP. **(B)** Size distribution of EVs obtained from astrocytes after the indicated treatments; traces were obtained by Nanoparticle Tracking Analysis (NTA) using a NanoSight instrument.

SUPPLEMENTAL MATERIAL AND METHODS

Nanoparticle Tracking Analysis for EVs

For Nanoparticle Tracking Analysis (NTA) to determine EV concentration and size, 300 µL of the EVs prepared in filtered PBS were measured on the NS300 instrument (NanoSight, Aumesbery, UK). NTA analytical software (NanoSight NTA version 3.2) was used for image capture and data analysis.

Additional RT-qPCR analysis from EVs

RT-qPCR validation of RNA in EVs was performed as indicated in the main manuscript. Additional amplicons were measured using the following primer pairs (each forward and reverse): TGCACCACCAACTGCTTAGC and GGCATGGACTGTGGTCATGAG for *GAPDH* mRNA, GCACAGAGCCTCGCCTT and GTTGTCGACGACGAGCG for *ACTB* mRNA, GTTACGGTCGGAGGCCG and GTGAGAGTGGCGGGGTC for *CDKN2A* mRNA, and AGTCAGTTCCTTGTGGAGCC and CATGGGTTCTGACGGACAT for *CDKN1A* mRNA.

Protein analysis for cell apoptosis

Whole-cell lysates and used for Western Blot as described in the main text. After size-fractionation by SDS-PAGE and transfer of whole-cell lysates, the blots were incubated with antibodies that recognized cleaved PARP (Santa Cruz Biotechnology), cleaved caspase 3, and GAPDH (Cell Signaling Technology).

Kim, Meng, et al. Supplemental Figure 1

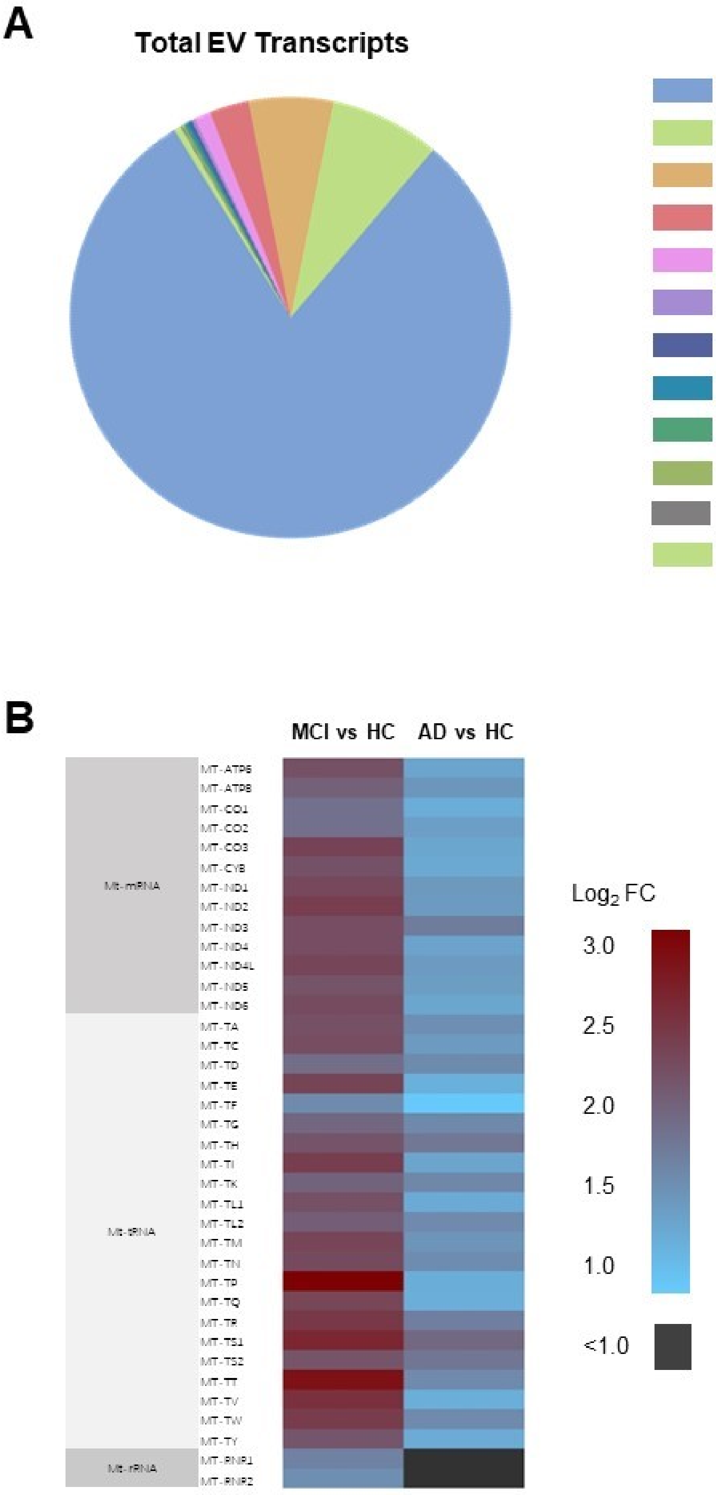

Kim, Meng, et al. Supplemental Figure 2

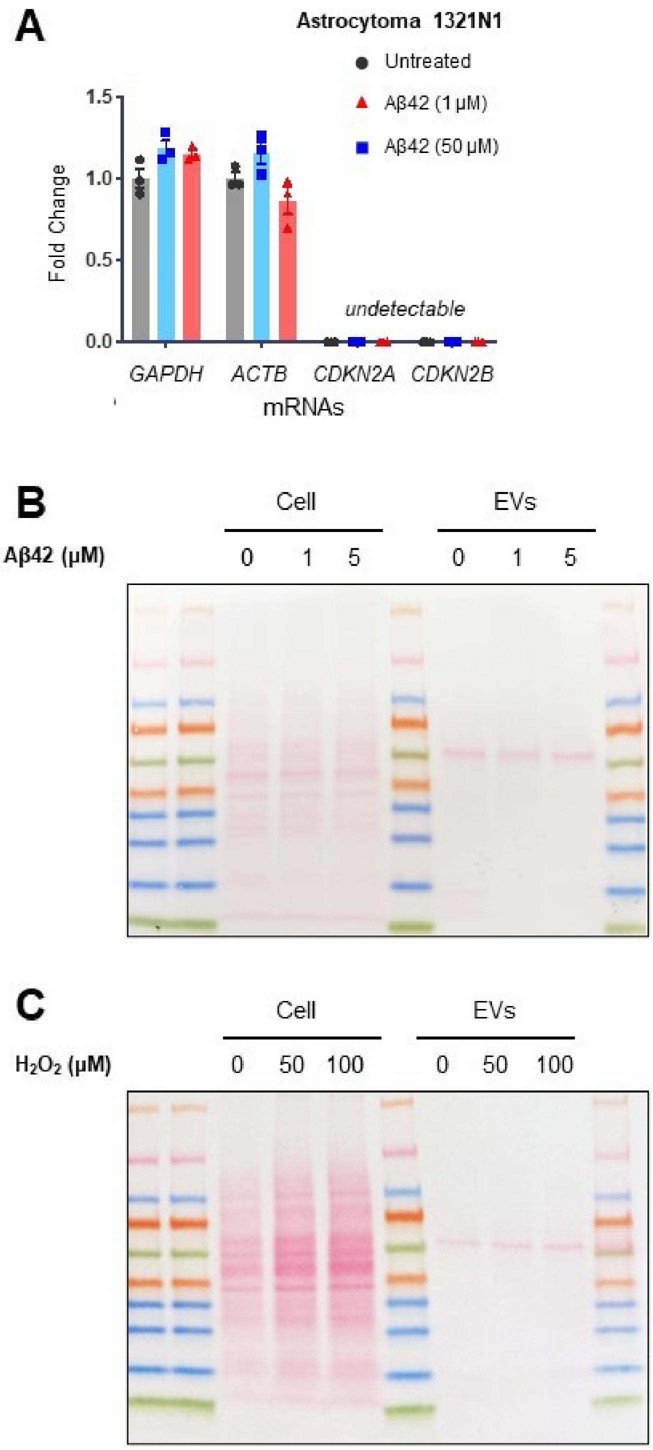

Kim, Meng, et al. Supplemental Figure 3

A

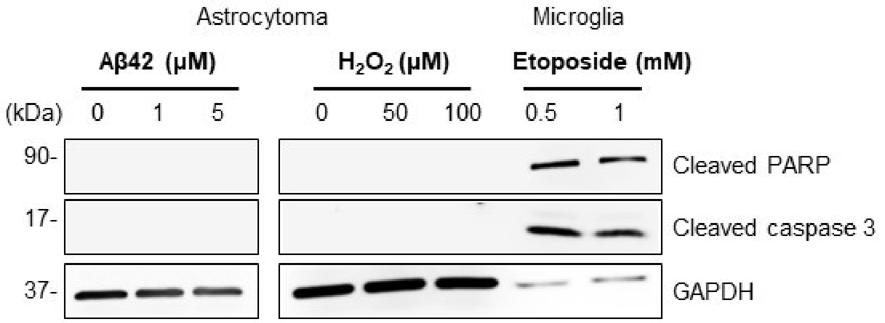

B

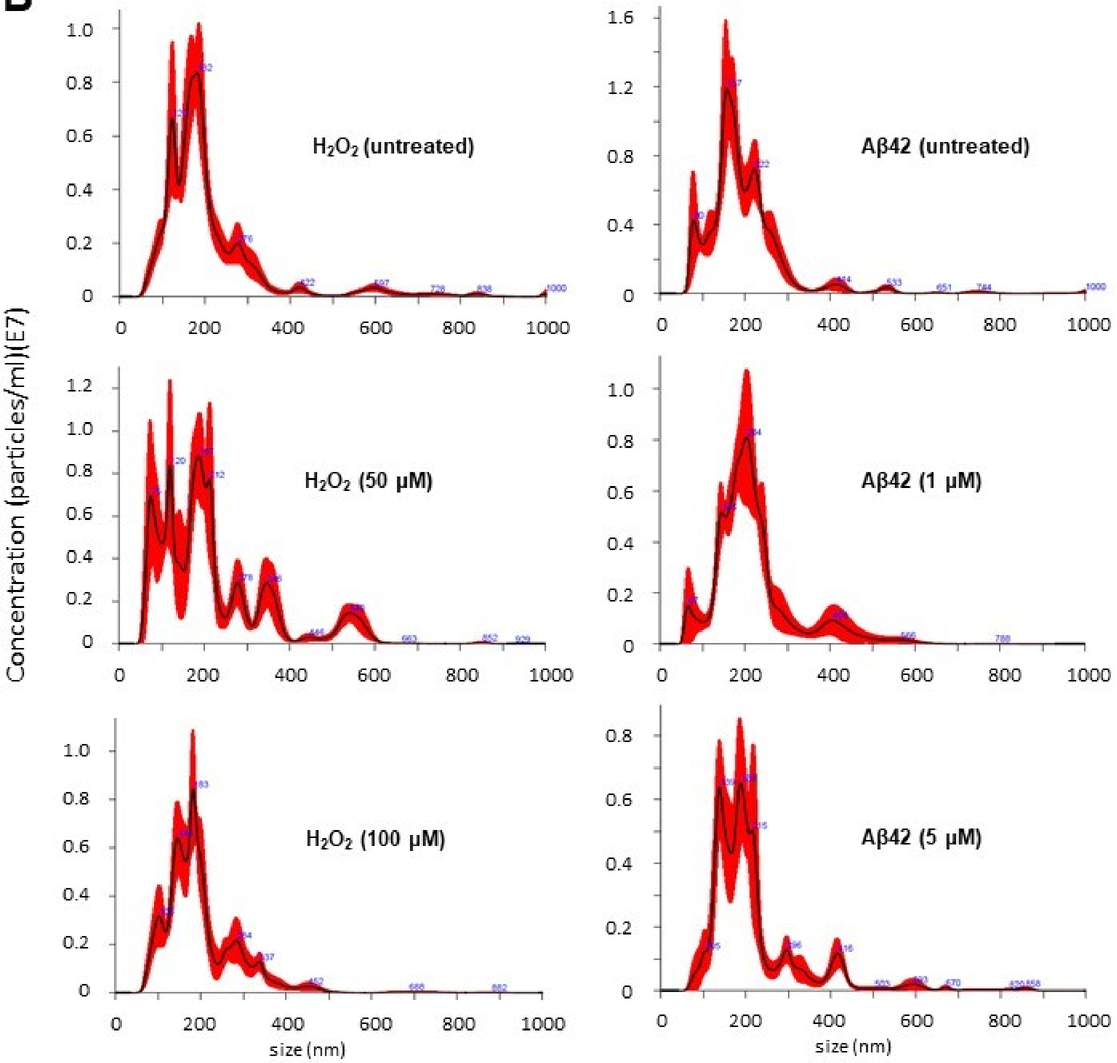

Supplement: Supplementary file 1 [file Data_Sheet_1.pdf]
